# Study on the differences between Hoek–Brown parameters and equivalent Mohr–Coulomb parameters in the calculation slope critical acceleration and permanent displacement

**DOI:** 10.1038/s41598-024-65494-3

**Published:** 2024-07-02

**Authors:** Cheng Li, Xi Zhao, Xingqian Xu, Xin Qu

**Affiliations:** 1 School of Architectural Engineering, Kaili University, Kaili, 556011 Guizhou China; 2 College of Water Conservancy, Yunnan Agricultural University, Kunming, 650201 Yunnan China; 3 School of Civil and Architecture Engineering, Anyang Institute of Technology, Anyang, 455000 Henan China

**Keywords:** Slope stability, Lower-bound of finite element limit analysis, HB strength criterion, MC strength criterion, Critical acceleration, Permanent displacement, Natural hazards, Solid Earth sciences

## Abstract

Mohr–Coulomb (MC) strength criterion has been widely used in many classical analytical expressions and numerical modeling due to its simple physical calculation, but the MC criterion is not suitable for describing the failure envelope of rock masses. In order to directly apply MC parameters to analytical expressions or numerical modeling in rock slope stability analysis, scholars established a criterion for converting Hoek–Brown (HB) parameters to equivalent MC parameters. However, the consistency of HB parameters and equivalent MC parameters in calculating critical acceleration of slope needs to be further explored and confirmed. Therefore, HB parameters are converted into equivalent MC parameters by considering the influence of slope angle (1# case and 2# case when slope angle is not considered and slope angle is considered respectively). Then, the lower-bound of finite element limit analysis is used for numerical modeling, and the results of calculating critical acceleration using HB parameters and equivalent MC parameters are compared, and the influence of related parameters on the calculation of critical acceleration is studied. Finally, the influence of different critical accelerations on the calculation of slope permanent displacement is further analyzed through numerical cases and engineering examples. The results show that: (1) In the 1# case, the critical acceleration obtained by the equivalent MC parameters are significantly larger than that obtained by the 2 #case and the HB parameters, and this difference becomes more obvious with the increase of slope angle. The critical acceleration obtained by the 2# case is very close to the HB parameters; (2) In the 1# case, slope height is inversely proportional to ΔAc (HB_(Ac) _− 1#_(Ac)_), and with the increase of slope height, ΔAc decreases, while in the 2# case, the difference of ΔAc (HB_(Ac)_  − 2#_(Ac)_) is not significant; (3) In the 1# case, the sensitivity of the HB parameters to ΔAc is *D* > *GSI* > *m*_*i*_ > *σ*_*ci*_, but in the 2# case, there is no sensitivity-related regularity; (4) The application of HB parameters and equivalent MC parameters in slope permanent displacement is studied through numerical cases and engineering examples, and the limitations of equivalent MC parameters in rock slope stability evaluation are revealed.

## Introduction

The advancement of the global economy and the rapid pace of urbanization have underscored the critical role of geotechnical engineering in the construction of infrastructure. Within this context, the analysis of slope stability has consistently been a focal point of research in geotechnical engineering, earthquake engineering, and engineering geology^1–4^. Previous studies have extensively explored various methods for slope stability analysis and have put forth a range of evaluation indicators and calculation models. Notably, the HB strength criterion and MC strength criterion have emerged as the most commonly utilized criteria, tailored for analyzing the stability of rock slope and soils slope, respectively. The MC strength criterion, in particular, offers a straightforward formula grounded in stress conditions and material characteristics, making it applicable to diverse soil types^5,6^. The key advantages of the MC parameters include its tangible physical interpretation, uncomplicated computation, and ease of comprehension and implementation^7^. However, when it comes to rock slopes, the presence of discontinuous and heterogeneous rock masses featuring intact rocks and natural or man-made discontinuities like joints, faults, bedding planes, and fractures complicates the analysis. Many commercial software programs and theoretical frameworks rely on shear strength parameters (cohesion and friction angles) to assess slope stability, neglecting the nonlinear nature of rock mass strength. Furthermore, the linear nature of the MC strength criterion often proves inadequate for delineating the failure envelope of rock masses^8^, and the indiscriminate use of MC parameters to characterize the properties of all slopes, whether rock or soil, may yield significant inaccuracies in stability calculations.

Over the past two decades, the generalized HB strength criterion has been effectively utilized across various rock types, establishing itself as the predominant strength criterion in rock slope engineering^9–11^. This criterion has addressed the limitations of the MC strength criterion when assessing rock slope stability. Previous research has delved into the distinctions between HB parameters and equivalent MC parameters in the context of slope stability analysis^12–18^. For instance, scholars such as Li^15^ and Deng^16^ have employed limit analysis and limit equilibrium methods to compare the impact of HB parameters versus equivalent MC parameters on calculating slope safety factors, respectively. Their findings indicate that for slope angles equal to or less than 45° and safety factors approaching 1, the disparity in safety factor calculations between HB and equivalent MC parameters is negligible. On the other hand, Zhao^17^ utilized HB and equivalent MC parameters to evaluate the permanent displacement of fractured rock slopes through upper-bound analysis and rigid block displacement techniques. The outcomes suggest that the MC strength criterion might overestimate the seismic stability of rock slopes compared to the HB strength criterion. Additionally, Chen and Lin^18^ utilized the gravity increase method to assess the variance between HB parameters and equivalent MC parameters in determining slope safety factors. Their results demonstrate that when safety factors are close to 1, HB parameters and equivalent MC parameters exhibit relatively minor differences, with the disparity in safety factor calculations gradually increasing as HB parameters undergo incremental adjustments.

Although HB parameters and MC parameters have been widely used in practical engineering, their differences in calculating the critical acceleration of slopes have not been fully studied, and there are still the following shortcomings: the main focus is on the applicability and accuracy of a single parameter, while the comparison and optimization of the two parameters in the slope stability analysis are rarely involved; focus on comparing the differences between the two parameters in calculating the slope safety factor, but lack of researches on the differences between HB parameters and equivalent MC parameters in calculating the critical acceleration of slopes. The critical acceleration of a slope is a crucial metric for evaluating its stability^19,20^, as it plays a significant role in predicting permanent displacement when combined with empirical displacement prediction models. In addition, the MC criterion is linear and has a small number of strength parameters. Many classic analytical equations and commercial codes are based on the MC model. Although the HB criterion is proposed as a non-linear scale, its parameter evaluation is subjective and therefore cannot be directly applied to analytical equations and commercial codes. Therefore, it is necessary to convert the HB strength parameters into the equivalent MC strength parameters for calculation. However, during the conversion process, many studies have overlooked the influence of slope angle, which will cause significant errors in the evaluation of slope stability. In summary, a thorough investigation into the differences between HB parameters and equivalent MC parameters in calculating critical acceleration not only enhances the rational and reliable assessment of slope stability but also contributes to advancing research in this area.

This study employs the lower-bound of finite element limit analysis to scrutinize the variations between critical accelerations computed using HB parameters and equivalent MC parameters under changing influencing factors such as HB parameters, slope height, and slope angle, as well as the sensitivity of individual HB parameters. Subsequently, the study compares the outcomes of different permanent displacements by integrating various critical accelerations with empirical displacement prediction models. Finally, the research delves into the discrepancies in critical acceleration calculations and their implications on slope stability analyses through engineering case studies.

## Calculation scheme and model

### Generalized Hoek–Brown strength criterion

E. Hoek and E.T. Brown derived a mathematical equation that characterizes the nonlinear failure behavior of rock formations based on an extensive series of experimental findings^21^. Following several iterations, Hoek^22^ introduced the generalized HB strength criterion (Eqs. 1–4):

1 $$\sigma_{1} = \sigma_{3} + \sigma_{c} \left( {m_{b} \frac{{\sigma_{3} }}{{\sigma_{c} }} + s} \right)^{a}$$

2 $$m_{b} = m_{i} \exp (\frac{GSI - 100}{{28 - 14D}})$$

3 $$s = \exp \left( {\frac{GSI - 100}{{9 - 3D}}} \right)$$

4 $$a = \frac{1}{2} + \frac{1}{6}\left[ {\exp ( - \frac{GSI}{{15}}) - \exp ( - \frac{20}{3})} \right]$$

where, *σ*_*1*_ and *σ*_*3*_ represent the maximum principal stress and the minimum principal stress, respectively, while *σ*_*c*_ denotes the uniaxial compressive strength. The material constant mi characterizes the hardness of the rock mass, and empirical parameters *m*_*b*_, *s*, and *a* are also considered. The values of *m*_*b*_ and *a* are specific to the type of rock mass, with *s* indicating the level of fracture within the rock mass. Additionally, *GSI* serves as the geological intensity index, and *D* accounts for the disturbance parameter related to blasting stress waves and excavation stress release.

To simplify the conversion process between the MC parameters and the HB parameters, Hoek^22^ proposed an equivalent conversion relationship (Eqs. 5–7). However, Li^15,23^ discovered that Hoek's conversion relationship may led to significant calculation errors when dealing with steep slopes (slope angle ≥ 45°). Consequently, adjustments to the slope angle parameters (*k* and *m*) were suggested (Eqs. 8–9) to enhance accuracy in such scenarios:

5 $$c^{\prime } = \frac{{\sigma_{ci} \left[ {\left( {1 + 2a} \right)s + \left( {1 - a} \right)m_{b} \sigma_{3n}^{\prime } } \right]\left( {s + m_{b} \sigma_{3n}^{\prime } } \right)^{a - 1} }}{{(1 + a)(1 + 2a)\sqrt {1 + \frac{{6am_{b} \left( {s + m_{b} \sigma_{3n}^{\prime } } \right)^{a - 1} }}{(1 + a)(1 + 2a)}} }}$$

6 $$\varphi^{\prime } = \sin^{ - 1} \left[ {\frac{{6am_{b} \left( {s + m_{b} \sigma^{\prime }_{3n} } \right)^{a - 1} }}{{2(1 + a)(1 + 2a)6am_{b} \left( {s + m_{b} \sigma^{\prime }_{3n} } \right)^{a - 1} }}} \right]$$

7 $$\sigma^{\prime }_{3n} = \frac{{\sigma^{\prime }_{3\max } }}{{\sigma_{ci} }}$$

8 $$\sigma^{\prime }_{cm} = \sigma_{ci} \frac{{\left[ {m_{b} + 4s - a\left( {m_{b} - 8s} \right)} \right]\left( {\frac{{m_{b} }}{4} + s} \right)^{a - 1} }}{2(1 + a)(2 + a)}$$

9 $$\frac{{\sigma^{\prime }_{3\max } }}{{\sigma^{\prime }_{cm} }} = k\left( {\frac{{\sigma^{\prime }_{cm} }}{\gamma H}} \right)^{m}$$

where, *σ′*_*3max*_ is the maximum upper limit of the maximum confining pressure, and *c’* is the cohesive force, *φ’* Is the internal friction angle, and *k* and *m* are parameters related to slope angle. For Eq. (9): when the influence of slope angle is not considered, *k* = 0.72 and *m* = − 0.91^22^; When considering the influence of slope angle^15^: when the slope angle < 45°, *k* = 0.41, *m* = − 1.23; When the slope angle is ≥ 45°, *k* = 0.2 and *m* = − 1.07. In addtion, this study adopts the non correlated flow rule for calculation, and the dilation angle is taken as 0°.

### Newmark model

The Newmark model is widely utilized in the assessment of slope permanent displacement due to its succinct mathematical formulation^24,25^. This model represents the slope as a rigid block and considers slope failure as the movement of this block sliding on a plane. The Newmark model can be described as:

10 $$FS = \frac{c}{\gamma t\sin \beta } + \frac{\tan \phi }{{\tan \beta }} - \frac{{n\gamma_{\omega } \tan \phi }}{\gamma \tan \beta }$$

11 $$a_{c} = (FS - 1)g\sin \beta$$

where, *FS* is the safety factor of slope; *c* is the cohesion, *γ* is the unit weight, *t* is the depth of the slope sliding surface, *a*_*c*_ is the slope critical acceleration, *g* is the gravitational acceleration, *β* is the slope angle, *φ* is the friction angle,* γ*_*w*_ is the unit weight of water, and *n* is related to groundwater.

It is important to highlight two key considerations: firstly, for the sake of simplicity, the study adopted the values *t* = 3 m and *n* = 0^24^. Secondly, the Newmark model necessitates the utilization of MC parameters (*c* and *φ*) for computation, while the approach employed in this article relies on the HB strength criterion for calculations. Consequently, in order to juxtapose the calculation outcomes derived from the Newmark model (utilizing equivalent MC parameters) and the HB parameters, it is imperative to employ a conversion mechanism to transform the parameters^15,23^.

### Numerical model

**Figure 1:**
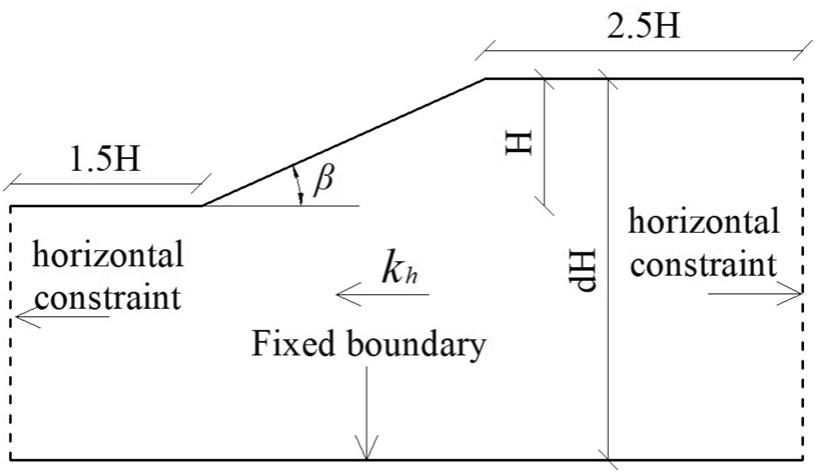
Generalized slope model.

In this investigation, a generalized slope model is employed for a detailed examination, as illustrated in Fig. 1. Within this model, the parameter *k*_*h*_ denotes the horizontal critical acceleration, while *d* signifies the depth coefficient. As per Loukidis et al.^26^, it is recommended that the value of *d* greater than or equal to 3. The bottom of the slope model is defined as a fixed boundary, with horizontal constrained boundaries applied on both sides. The lower-bound solution and upper-bound solution of finite element limit analysis are suitable for slope design and understanding of slope failure mechanism respectively^27^. As the focus of this study is to compare the difference between HB parameters and equivalent MC parameters in calculating slope critical acceleration, rather than to analyze the failure mechanism of slope, the lower bound solution method is adopted in this study. The computational software employed in this process is OptumG2 (academic version)^28^.

**Figure 2:**
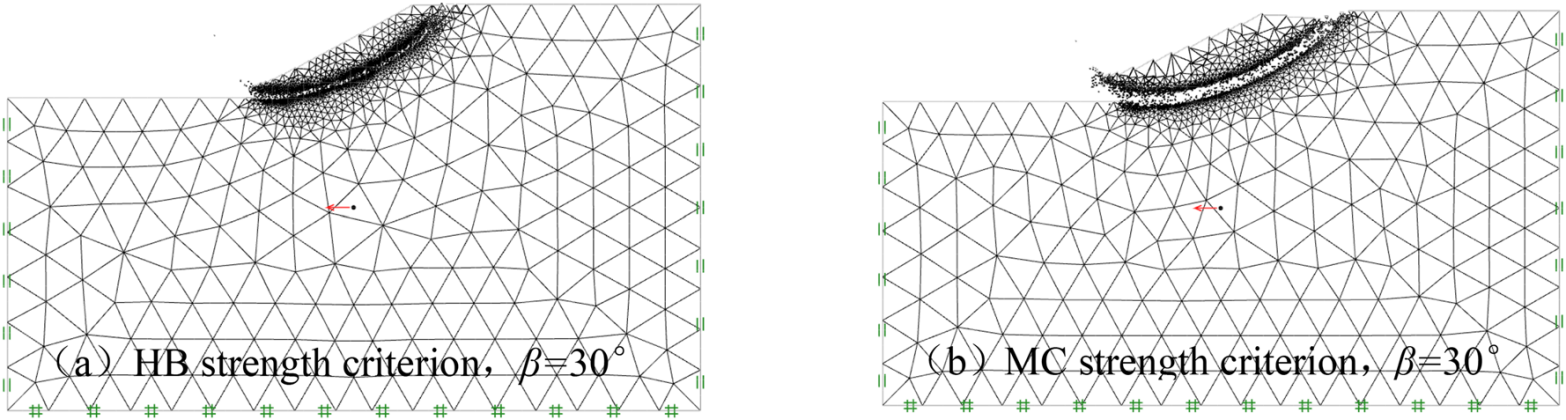
Calculation results of sliding surface.

During the establishment of the numerical model, an adaptive mesh generation technique is utilized, with the adaptive iteration number set to 3. The initial number of elements in the model is 2000, with a maximum of 4000 elements allowed^29–31^. The primary controlling parameter for adaptive mesh generation is shear dissipation. Furthermore, the mesh refinement factor is designated as 0.25, and the mesh roughness factor is set at 1.50. The outcomes of the calculations pertaining to the sliding surface and the boundary conditions are depicted in Fig. 2. Note that Fig. 2 is only a dynamic result derived by OptumG2 for easy viewing of sliding surfaces and failure modes, and has no strictly physical meaning.

Table 1 provides a detailed overview of the slope model's fundamental parameters, including slope angle, slope height, and strength parameters. Table 2 displays the HB parameters and their corresponding equivalent MC parameters, which are essential for assessing slope stability. To investigate the impact of parameter variations on slope stability, this study employs a single-factor analysis method to adjust HB parameters based on predetermined rates of change. The corresponding equivalent MC parameters are also computed to ensure numerical calculation accuracy. The change ranges and change amplitude of of these parameters are documented in Table 2 for further analysis and discussion. Taking into account the changes in HB parameters and equivalent MC parameters while keeping other conditions constant, the critical acceleration of the slope for each parameter combination listed in Table 2 is determined using finite element limit analysis. This approach allows for a deeper understanding of how parameter adjustments affect slope stability, offering valuable theoretical insights for practical engineering applications.

**Table 1:** Basic parameters of slope model.

*γ*(kN/m^3^)	*β*(°)	*H*(m)	*σ*_*ci*_(MPa)	*GSI*	*m*_*i*_	*D*
22	30, 60	10,20,30	10	20	10	0.5

The variation spacing of *σ*_*ci*_ is 2Mpa; the variation spacing of *GSI* and *m*_*i*_ is 2; the variation spacing of *D* is 0.1

**Table 2:** HB calculation parameters and corresponding equivalent MC calculation parameters.

H(m)	HB parameter range	1# equivalent *c*(kPa)	1# equivalent *φ*(°)	2# equivalent *c*(kPa)	2# equivalent *φ*(°)
10	*σ*_*ci*_(10–30 MPa)	31.31–48.09	32.65–40.38	18.11–23.48	38.95–49.03
	*GSI*(10–30)	18.24–44.9	25.19–38.30	11.66–24.89	29.43–45.74
	*m*_*i*_(10–30)	31.34–46.38	32.65–42.49	18.11–22.47	38.95–50.07
	*D*(0–1)	46.15–13.97	41.10–17.63	24.54–10.26	48.44–20.56
20	*σ*_*ci*_(10–30 MPa)	48.08–73.24	27.91–35.5	32.15–39.24	32.36–42.78
	*GSI*(10–30)	28.24–68.04	21.3–33.19	21.11–42.52	23.86–38.98
	*mi*(10–30)	48.04–72.86	27.91–37.56	32.15–41.55	32.36–43.67
	*D*(0–1)	70.79–20.81	36.2–14.24	42.36–17.66	42.09–15.58
30	*σ*_*ci*_(10–30 MPa)	61.58–94.01	25.25–32.65	45.08–54.34	28.6–38.95
	*GSI*(10–30)	36.37–86.88	19.19–30.25	29.8–58.98	20.85–34.93
	*mi*(10–30)	61.58–94.65	25.25–34.68	45.08–59.45	28.6–39.8
	*D*(0–1)	91.13–26.19	33.34–12.48	59.27–24.14	38.23–13.09

## Result analysis

### Differences between HB parameters and equivalent MC parameters

**Figure 3:**
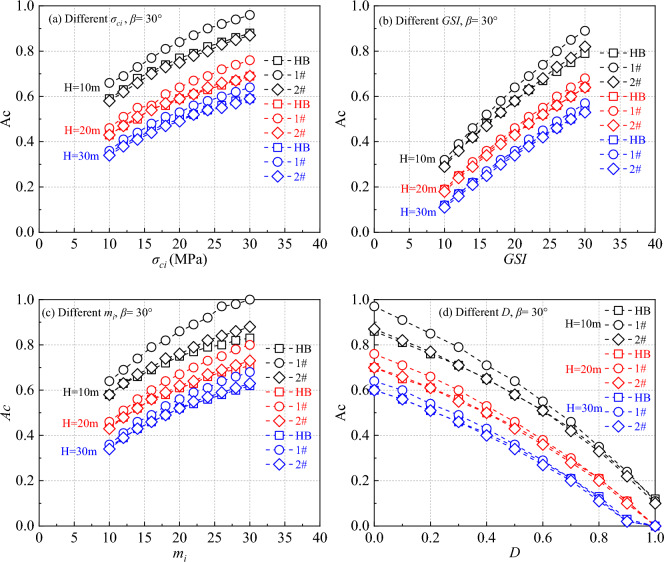
The calculation results of critical acceleration for different parameters when the slope is 30°.

**Figure 4:**
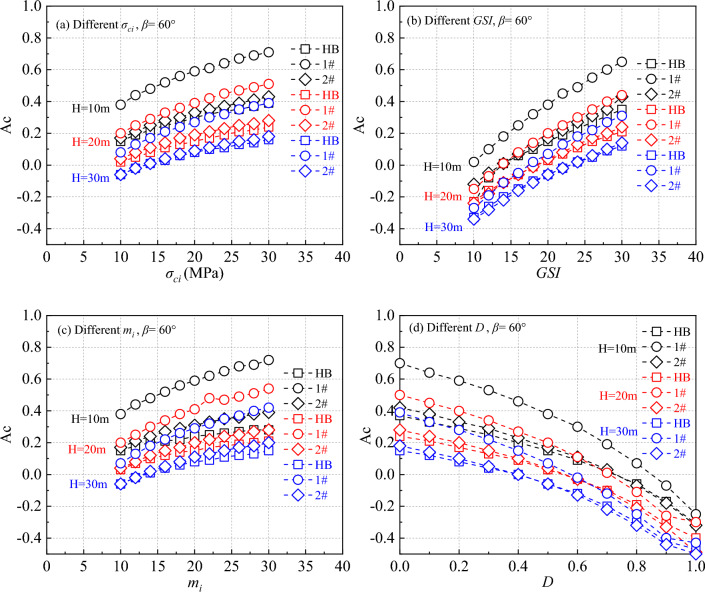
The calculation results of critical acceleration for different parameters when the slope is 60°.

**Figure 5:**
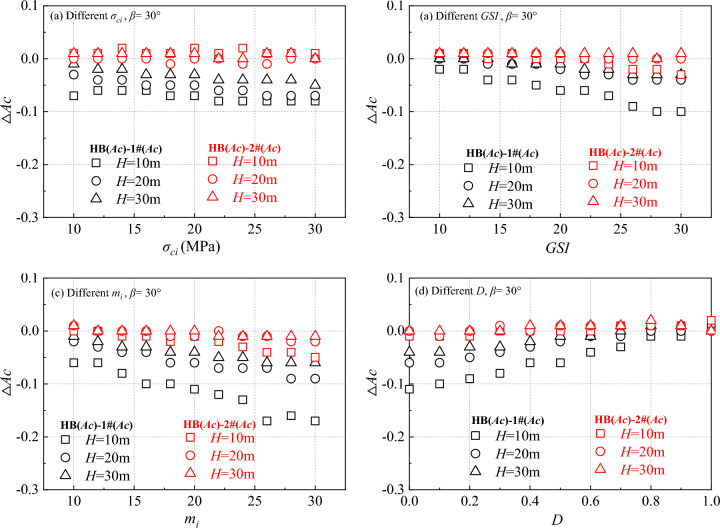
Calculation results of different ΔAc at H = 10 m, 20 m and 30 m when the slope is 30°

**Figure 6:**
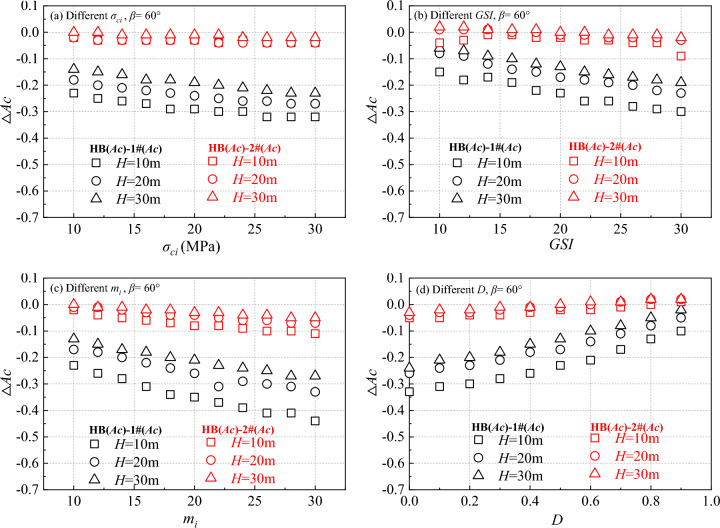
Calculation results of different ΔAc at H = 10 m, 20 m and 30 m when the slope is 60°

According to the proposed calculation scheme, a detailed numerical simulation of a generalized slope model was conducted using the lower-bound of finite element limit analysis. Through Eqs. (1–9), the equivalent cohesion *c* and internal friction angle *φ* were calculated for various HB parameters. The critical acceleration of the slope under different parameter configurations was compared and analyzed, with the results presented in Figs. 3, 4. It is worth noting that in Fig. 3, when one strength parameter changes, other strength parameters remain unchanged as shown in Table 1. For example, in Fig. 3a, the range of variation of σ_ci_ is from 10 to 30 MPa, while GSI, m_i_, and D are equal to 20,10 and 0.5, respectively. The same applies to Figs. 3b–d, 4, 5, 6.

Figure 3 illustrates the variation in critical acceleration with changing *H* for different HB parameter settings at a slope angle of 30°. Figure 4 shows the impact of *H* changes on critical acceleration as the slope angle increases to 60°. These figures clearly demonstrate that the critical acceleration of the slope is positively related to *σ*_*ci*_, *GSI*, and *m*_*i*_, and inversely related to the *D*. The critical acceleration obtained in 2# case closely aligns with the critical acceleration obtained using the HB parameters.

Further examination of Fig. 3 reveals that in the low critical acceleration category, the HB parameters align closely with the results obtained in the 1# case. For instance, in Fig. 3b, when the *H* is 30 m and the *GSI* is 10, and in Fig. 3d, when the *H* is 30 m and the *D* is 1.0. However, as the values of *σ*_*ci*_, *GSI*, *m*_*i*_ increase, and *D* decreases, the disparity between the calculated results of the two parameters gradually becomes more pronounced. This trend is in line with the findings of Chen and Lin when determining the safety factor^12^. When the *β* is raised to 60°, as depicted in Fig. 4, the contrast between the HB parameters and the results from the 1# case becomes even more significant.

### Influence of H on ΔAc

In the field of slope stability analysis, the *β* and *H* are crucial factors that directly influence the calculation of critical acceleration. Specifically, when *β* is set at 30° and 60°, the impact of *H* on critical acceleration calculation is significant, as illustrated in Figs. 5, 6. The difference in critical acceleration, denoted as ΔAc, is calculated using HB parameters and equivalent MC parameters.

When examining the influence of *β* on critical acceleration, changes in *H* have a minor effect on the difference (HB_(Ac)_-2#_(Ac)_). However, without considering the *β*, alterations in *H* have a more pronounced impact on the critical acceleration difference (HB_(Ac)_-1#_(Ac)_), with larger *β* leading to greater differences. Further investigation revealed that irrespective of *H*, the critical acceleration difference (HB_(Ac)_-1#_(Ac)_) exhibited a gradual increase as *GSI*, *m*_*i*_, *σ*_*ci*_ increased and the *D* decreased.

**Figure 7:**
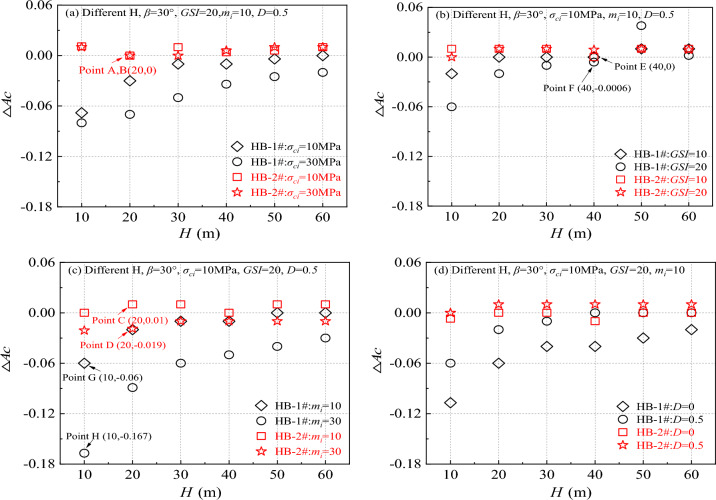
Calculation results of different ΔAc when *H* increases from 10 to 60 m.

To further explore the relationship between the change in ΔAc and the change in *H*, this study gradually increased the *H* from 10 to 60 m in increments of 10 m while maintaining a fixed slope angle of 30 degrees. The study meticulously calculated the ΔAc corresponding to each *H* increment. To comprehensively analyze the impact of intensity parameters on the results, specific combinations of HB parameters were selected, including *σ*_*ci*_ values of 10 and 30 MPa, *GSI* values of 10 and 20, *m*_*i*_ values of 10 and 30, and *D* values of 0 and 0.5, resulting in four distinct sets of calculation models. The outcomes of these models were meticulously recorded and utilized for further examination, with detailed results presented in Fig. 7.

The analysis from Fig. 7 reveals that when considering the influence of *β* (HB_(Ac)_-2#_(Ac)_), the differences in ΔAc values calculated using various strength parameter combinations ((*σ*_*ci*_, *GSI*, *m*_*i*_, D) are not substantial. The differences mainly hover around 0, with minimal fluctuations, ranging from a minimum of 0 (as seen in Fig. 7a) to a maximum of 0.009 (as shown in Fig. 7c). However, when not factoring in the influence of *β* (HB_(Ac)_-1#_(Ac)_), the disparities in ΔAc values calculated with different strength parameter combinations increase significantly. The differences range from 0.0006 to 0.107, indicating a broader range of variation.

Further investigation revealed that as the *H* increased, ΔAc exhibited a gradual decrease. Additionally, it was observed that the magnitude of HB parameters (*σ*_*ci*_, *GSI*, *m*_*i*_) was positively correlated with the differences in ΔAc, meaning that higher HB parameter values led to greater differences and rates of change. The impact of *D* was such that smaller *D* values resulted in lower disturbance parameters and larger ΔAc. These findings offer a crucial theoretical foundation for engineers to comprehend and forecast slope stability.

### The sensitivity analysis of HB parameters to ΔAc

Given that HB parameters and equivalent MC parameters do not align perfectly, it is important to examine the impact of HB parameters such as *σ*_*ci*_, *GSI*, *m*_*i*_, and *D* on ΔAc. Figures 5, 6 illustrate that each HB parameter affects critical acceleration differently, resulting in variations in ΔAc. To better understand the extent and sequence of these influences, the relative deviation ratio is employed. The sensitivity of HB parameters to the influence of ΔAc can be determined using the following analytical formula:

12 $$\delta = \frac{{\delta_{m\Delta Ac} }}{{\delta_{{D_{m} }} }} = \frac{{(D_{m\Delta Aci} - D_{m\Delta Ac} )D_{m\Delta Ac} }}{{(D_{mi} - D_{m} )D_{m} }}$$

In Eq. (12), *D*_*m*_, *D*_*mΔAc*_, *D*_*mi*_, and *D*_*ΔAci*_ represent the reference values and rates of change of HB parameters and ΔAc, respectively. This formula allows for a quantitative analysis of the impact of HB parameters on ΔAc and a better understanding of how each HB parameter contributes to the change in ΔAc.

In this section, specific values were assigned for *D*_*k*_: *σ*_*ci*_ = 20 MPa, *GSI* = 20, *m*_*i*_ = 20, and *D* = 0.5. To illustrate Eq. (12), the variable *GSI* is used as an example. For instance, when *H* = 10 m (in the 1# case), *GSI* = 10 is the reference value *D*_*GSI*_, and the relative *D*_*GSI*ΔAc_ = − 0.02. Subsequently, *GSI* increases to 20 MPa with *D*_*GSI*ΔAc_ = − 0.06. This means that *δD*_*GSI*_ = (20–10)/10 × 100% = 50%, and *δD*_*GSI*ΔAc_ = (− 0.06−(− 0.02))/(− 0.02) × 100% = 200%. In other words, the change in ΔAc is 200% when the change in *GSI* is 50%. This pattern is consistent with the other three parameters.

**Figure 8:**
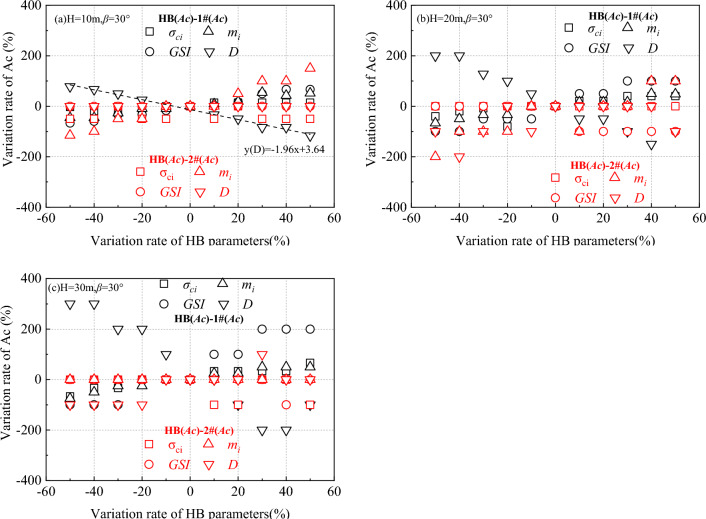
Sensitivity of Ac Variation at H = 10 m, 20 m and 30 m when the slope is 30°

According to Eq. 12, the rate of change curves for the ΔAc and HB parameters are plotted, as depicted in Fig. 8. To conduct a more detailed comparison of the sensitivity of each intensity parameter, a fitted straight line for each parameter is also included, shown in the y(D) section of Fig. 8a. The absolute values of the slopes of these fitted lines accurately represent the sensitivity of the HB parameters, as detailed in Table 3. Analysis of Table 3 reveals that when not considering the impact of *β* (HB_(Ac)_-1#_(Ac)_), the maximum and minimum values of the *D* curve are respectively larger and smaller than those of the other curves. This observation clearly indicates that the slope of the *D* curve is the most significant. Similarly, the slopes of the *GSI*, *m*_*i*_, and *σ*_*ci*_ curves decrease sequentially. Therefore, when considering all factors, the slope ranking of the intensity parameter curve is *D* > *GSI* > *m*_*i*_ > *σ*_*ci*_, indicating that the sensitivity ranking is *D* > *GSI* > *m*_*i*_ > *σ*_*ci*_. This demonstrates that *D* has the most pronounced impact on ΔAc, followed by GSI and then mi, while σci has a relatively minor effect. This ranking differs from that of previous researchers when calculating the safety factor. However, when considering the influence of *β* (HB_(Ac)_-2#_(Ac)_), the fixed regularity in the slope ranking of each intensity parameter is lost.

**Table 3:** The slope (sensitivity) of each fitted line in Fig. 8

*β*(°)	*H*(m)	Type of △*Ac*	*σ*_*ci*_(MPa)	*GSI*	*m*_*i*_	*D*
30	10	HB(*Ac*)-1#(*Ac*)	0.37	1.39	1.05	− 1.96
		HB(*Ac*)-2#(*Ac*)	− 0.04	0	2.52	0
	20	HB(*Ac*)-1#(*Ac*)	0.91	2.32	1.13	− 3.62
		HB(*Ac*)-2#(*Ac*)	0	− 1.36	2.45	1.09
	30	HB(*Ac*)-1#(*Ac*)	1.12	3.54	1.25	− 5.36
		HB(*Ac*)-2#(*Ac*)	− 0.72	− 0.36	0	1.54

### Influence of different critical accelerations on permanent displacement

In general, there are two main methods used to assess the seismic stability of slopes: the safety factor method and the permanent displacement method. Engineers often prefer the permanent displacement method for quickly evaluating slope seismic stability due to its straightforward concept, easy calculations, and ability to bypass many preprocessing steps. To determine permanent displacement, it is crucial to calculate the critical acceleration of the slope and select a suitable empirical displacement prediction model.

The empirical displacement prediction model relies on statistical analysis of numerous actual seismic accelerations^32^. It offers a rapid and uncomplicated approach to predicting the permanent displacement of a slope under earthquake conditions, regardless of the ground motion's time history. Therefore, during the project's initial design and rapid evaluation stages, it is advisable to assess the slope's seismic performance (permanent displacement) using empirical prediction models^33^:

(1)The empirical displacement prediction model proposed by Ambraseys and Menu is as follows^32^:

13 $$\lg D_{n} = 0.90 + \log \left[ {\left( {1 - \frac{{A_{c} }}{{A_{\max } }}} \right)^{2.5} \left( {\frac{{A_{c} }}{{A_{\max } }}} \right)^{ - 1.09} } \right] \pm 0.30$$

(2)The empirical displacement prediction model proposed by Jibson is as follows^33^:

14 $$\lg D_{n} = 0.215 + \log \left[ {\left( {1 - \frac{{A_{c} }}{{A_{\max } }}} \right)^{2.341} \left( {\frac{{A_{c} }}{{A_{\max } }}} \right)^{ - 1.438} } \right] \pm 0.510$$

**Figure 9:**
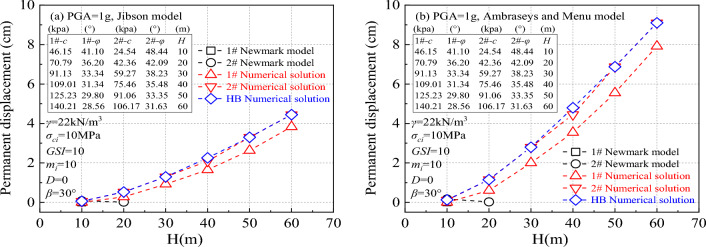
Influence of different critical accelerations on permanent displacement.

Figure 9 illustrates the variations in permanent displacement calculations based on different critical accelerations, taking into account the influence of *β* and different *H*. The parameters relevant to slope stabilization in Fig. 9 include PGA = 1.0 g, *σ*_*ci*_ = 10Mpa, *GSI* = 10, *m*_*i*_ = 10, *D* = 0, *γ* is 22kN/m^3^, *β* = 30°. The results in Fig. 9 demonstrate that regardless of the empirical displacement prediction model used, the outcomes align closely with the numerical solution obtained in this study. However, the Newmark model significantly underestimates the calculated results.

When determining the critical acceleration with the consideration of slope angle effect (2# numerical solution), the resulting permanent displacement aligns well with the HB numerical solution. Conversely, if the critical acceleration is calculated without factoring in the slope angle effect (1# numerical solution), the resulting permanent displacement significantly underestimates the slope hazard. Notably, as the *H* increases gradually, the disparity between the permanent displacement calculated without considering the slope angle effect and the numerical solution also grows. Moreover, for *H* values between 10 and 20 m, the critical acceleration computed by the Newmark model is excessively high, leading to the inability to determine the slope's permanent displacement. This limitation arises from the constraint that (1-*A*_*c*_/*A*_*max*_) cannot be negative, restricting the Newmark model's applicability within this range. Therefore, when utilizing the Newmark model to assess the slope's permanent displacement, it is crucial to select parameters within the appropriate range.

The permanent displacement predicted by the Ambraseys and Menu model^23^ exceeds that of the Jiobson model^22^. This discrepancy is due to the fact that the empirical displacement prediction model is based on numerous statistical laws derived from historical data analysis, which may not fully suit the specific conditions of individual slopes. Different empirical displacement prediction models yield varying results when estimating permanent displacement. Hence, when employing a permanent displacement model to evaluate slope stability, engineers must meticulously choose an empirical displacement prediction model that fits the slope's environment to ensure the accuracy and reliability of the assessment outcomes.

## Case analysis

The two selected rock slope cases discussed in this paper hold unique research value^8^. The Zipingpu reservoir rock slope, situated north of the reservoir in Sichuan Province, China, has a complex rock structure and stability concerns. The Huangnigang landslide, resulting from a rock slope failure during the Wenchuan earthquake in May 2008 in Chengdu, China, is of significant research importance.

Table 4 presents relevant parameters and calculation results. It shows that the critical acceleration obtained in the first case without considering slope angle greatly exceeds the critical acceleration calculated using HB parameters, leading to an overestimation of slope stability. However, in the second case where slope angle is considered, the critical acceleration aligns more closely with the HB parameter calculation, aiding engineers in more accurate slope stability evaluations.

**Table 4:** Relevant parameters and calculation results of two cases.

Parameters/calculation results	ZZP slope	HNG landslide
*σ*_*ci*_(Mpa)	100	50
*GSI*	40	17
*m*_*i*_	10	18
*γ*(kN/m^3^)	28	24
*H*(m)	100	140
*β(***°)**	50	53
1#equivalent *c*(kPa)	943.12	516.84
1#equivalent *φ*(°)	46.82	37.18
2#equivalent *c*(kPa)	470.78	197.95
2#equivalent *φ*(°)	57.28	47.33
HB(*Ac*)	0.89	0.31
1#(*Ac*)	1.19	0.56
2#(*Ac*)	0.93	0.35

For instance, in the ZZP slope case, the difference between critical acceleration and that calculated by HB parameters is 25.21% without considering slope angle, but this reduces to 4.30% when slope angle is factored in. Similarly, for the Huangnigang landslide, the difference is 44.64% without considering slope angle and 11.43% when slope angle is considered. In conclusion, analyzing these cases highlights the importance of fully considering slope angle when converting HB parameters to MC parameters for more precise results. This insight is crucial for future slope stability analysis and prevention efforts.

**Figure 10:**
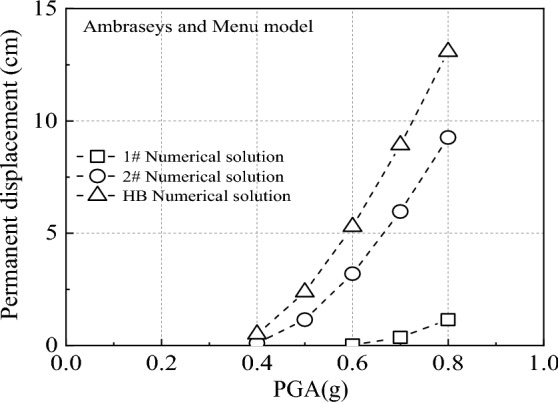
The permanent displacement of HNG landslide when the PGA gradually increases.

This study focuses on examining how different scenarios impact the calculation of permanent displacement, using HNG landslides as the subject of research. The analysis in Fig. 10 reveals that in the 1# case, there is a notable disparity between the calculated permanent displacement and that derived using the HB parameter. Particularly, at a PGA of 0.8 g, the difference peaks at 1144.49% [(13.08–1)/13.08 × 100%]. This outcome underscores the unreliability of using the MC parameters in the 1# case to determine permanent displacement and assess slope stability. It should be noted that the HNG landslide is located in Yingxiu Town, the epicenter of the 2008 Wenchuan earthquake. According to the China Earthquake Administration, the closest strong seismic station to the epicenter is Wolong Station in Wenchuan, Sichuan, which is 22.2 km away from the epicenter. The PGA is 0.98 g. Therefore, the range of PGA used in Fig. 10 is 0.4 g to 1.0 g.

Conversely, while variations persist between the permanent displacement in the 2# case and that calculated with HB parameters, in certain instances, this difference can still serve as a benchmark for evaluating slope stability. Building on prior research findings^16,21^, the paper defines slopes with permanent displacement equal to or exceeding 5 cm as unstable. In Fig. 10, when PGA is below 0.6 g, both the results in the 2# case and the permanent displacement calculated with HB parameters are under 5 cm, indicating slope stability. Conversely, when PGA is 0.7 g or higher, both the 2# case results and HB parameter calculations exceed 5 cm, signaling slope instability. This discovery offers a more precise basis for assessing slope stability, holding significant implications for slope evaluation.

## Conclusion

In the assessment of rock slope stability, albeit the MC strength criterion is extensively utilized in engineering practice, the HB criterion possesses evident advantages in mimicking the nonlinear behavior of rock. This study compares and scrutinizes the disparity between the HB parameters and the equivalent MC parameters in computing the critical acceleration of the slope, and extracts the following insights: When transforming the HB parameters to the equivalent MC parameters for recalculation, the impact of the slope angle should be acknowledged. This perspective has been substantiated as follows:

1. In the 1# case, unless the critical acceleration of the slope is relatively small, the critical acceleration calculated by the HB parameters is not consistent with the equivalent MC parameters. The critical acceleration estimated by the 2# case is remarkably close to the critical acceleration derived by the HB parameters.
2. In the 1# case, when the extent of slope height is 10–60 m, with the augmentation of slope height, ΔAc manifests a precipitous downward trend, and ΔAc is negatively correlated with slope height. In the 2# case, the discrepancy of ΔAc is negligible, and the absolute value of the difference is chiefly confined around 0, and the fluctuation amplitude is minor.
3. In the 1# case, the attained sensitivity order is: *D* > *GSI* > *m*_*i*_ > *σ*_*ci*_, and the HB parameters are linearly associated with the rate of ΔAc change, and the rate of ΔAc change is positively correlated with *GSI*, *m*_*i*_, *σ*_*ci*_, and negatively correlated with *D*. In the 2# case, there is no discernable regularity related to sensitivity;
4. Through numerical exemplifications (slope angle = 30°) and HNG landslide (slope angle = 53°), it can be observed that the permanent displacement realized in the 1# case deviates significantly from the permanent displacement achieved under the conditions of HB numerical resolution. When slope angle = 30°, the permanent displacement estimated in the 2# case is fundamentally the same as the permanent displacement realized under the stipulations of HB strength parameter. When slope angle = 53°, the permanent displacement uncovered in the 2# case differs from the permanent displacement realized under the conditions of HB numerical resolution. Nonetheless, it can still furnish some guidance for slope seismic stability analysis.

## Data Availability

All data generated or analysed during this study are included in this published article.
